# *Drosophila melanogaster* as a Model for Autism Spectrum Disorder: Insights into Lifelong Neuronal Vulnerability

**DOI:** 10.3390/ijms27188129

**Published:** 2026-09-12

**Authors:** Angelina Palacios-Muñoz

**Affiliations:** 1 Laboratorio de Genética y Conducta, Facultad de Odontología, Universidad de Valparaíso, Valparaíso 2360004, Chile; angelina.palacios@uv.cl; 2 Centro Interdisciplinario de Neurociencia de Valparaíso (CINV), Universidad de Valparaíso, Valparaíso 2340000, Chile; 3 Centro de Investigación en Ciencias Odontológicas y Médicas (CICOM), Facultad de Odontología, Universidad de Valparaíso, Valparaíso 2360004, Chile

**Keywords:** autism spectrum disorder, aging, neuronal vulnerability, mitochondrial homeostasis, neuroimmune signaling, circadian biology, proteostasis

## Abstract

Autism spectrum disorder (ASD) is a neurodevelopmental condition characterized by impaired social communication, restricted interests, and repetitive behaviors. Although traditionally considered a disorder of early brain development, growing evidence indicates that many ASD-associated genes continue to regulate neuronal physiology throughout adulthood. These genes participate in conserved cellular processes involved in synaptic organization, mitochondrial homeostasis, calcium signaling, proteostasis, intracellular trafficking, neuroimmune regulation, and circadian biology, many of which are also implicated in brain aging. This convergence supports the concept that developmental alterations can influence neuronal function long after neural circuits have formed. In this context, the fruit fly *Drosophila melanogaster* (*Drosophila*) has emerged as a powerful translational model for investigating the conserved mechanisms linking neurodevelopment and aging. Its high genetic conservation with humans, sophisticated neurogenetic tools, short lifespan, and robust behavioral assays enable longitudinal examination of developmental genetic alterations within a single experimental organism, facilitating mechanistic studies of neuronal function, stress adaptation, and age-dependent behavioral phenotypes. This review examines ASD from the perspective of lifelong neuronal vulnerability, highlighting how genetically diverse ASD-associated genes converge on common biological mechanisms that remain active beyond development. Furthermore, I discuss how *Drosophila* has advanced our understanding of these conserved pathways and provides a unique experimental platform for investigating how early developmental alterations shape neuronal function across the lifespan.

## 1. Introduction

Autism spectrum disorder (ASD) is a heterogeneous neurodevelopmental condition characterized by persistent deficits in social communication and social interaction together with restricted, repetitive patterns of behavior, interests, or activities as defined by the Diagnostic and Statistical Manual of Mental Disorders, Fifth Edition (DSM-5) [1,2]. Although ASD is typically diagnosed during early development, it is increasingly recognized as a lifelong condition whose biological consequences extend well beyond neurodevelopment [3,4,5,6,7]. Current epidemiological estimates indicate that ASD affects approximately 1–2% of the global population [8], and the growing number of autistic adults has shifted attention toward understanding how developmental alterations influence brain function throughout life. In addition to core behavioral features, adults with ASD show increased prevalence of anxiety, epilepsy, sleep disturbances, metabolic dysfunction, immune dysregulation, and premature mortality [9,10,11], suggesting that ASD involves persistent alterations affecting both neuronal and systemic physiology rather than processes restricted to early brain development.

Advances in next-generation sequencing (NGS), particularly whole-exome sequencing (WES) and whole-genome sequencing (WGS), have transformed our understanding of ASD biology by revealing an unexpectedly complex genetic architecture [12,13]. Hundreds of rare de novo variants, inherited mutations, copy number variants (CNVs), and common polygenic risk alleles have now been associated with ASD susceptibility [4,12,14,15,16,17]. Altogether, these studies indicate that ASD-associated genes participate in a relatively limited number of conserved biological pathways involved in synaptic organization, chromatin remodeling, RNA metabolism, calcium signaling, mitochondrial function, intracellular trafficking, cytoskeletal dynamics, and local protein translation [4,14,16,17,18,19].

The Simons Foundation Autism Research Initiative (SFARI) maintains the SFARI Gene database, one of the most widely used curated resources for ASD genetics research (SFARI; https://gene.sfari.org) [20]. The database integrates evidence from sequencing studies, copy number variant analyses, genome-wide association studies, and functional data to prioritize ASD-associated genes. Genes are classified according to the strength of the available evidence as Category 1 (High Confidence), Category 2 (Strong Candidate), or Category 3 (Suggestive Evidence). In addition, syndromic genes (S) are distinguished from non-syndromic ASD genes. Syndromic genes are those in which ASD occurs as part of a broader genetic syndrome accompanied by additional neurological or systemic manifestations, whereas non-syndromic genes are primarily associated with idiopathic ASD in the absence of a recognizable syndrome. Importantly, syndromic genes may also receive evidence-based rankings (e.g., 1S, 2S, or 3S) when independent data support their involvement in non-syndromic ASD. This classification framework facilitates candidate-gene prioritization, variant interpretation, and the identification of conserved biological pathways underlying the remarkable genetic heterogeneity of ASD.

Although ASD has one of the strongest genetic components among neurodevelopmental disorders, it is widely recognized as a multifactorial condition in which genetic susceptibility interacts with prenatal, perinatal, epigenetic, and environmental factors to influence disease risk and phenotypic variability [21,22,23,24]. These interactions are thought to contribute substantially to the clinical heterogeneity of ASD, helping explain why individuals carrying variants in the same gene may differ in symptom severity, developmental trajectory, and associated comorbidities.

One of the most important conceptual advances emerging from these genomic studies is the recognition that many ASD-associated genes should no longer be viewed exclusively as regulators of early brain development. Genes such as *SHANK3*, *NRXN1*, *FMR1*, *PTEN*, and *TRPC6*, among many others, have functions that extend beyond early neurodevelopment, and their encoded proteins continue to regulate synaptic remodeling, mitochondrial quality control, autophagy, calcium signaling, intracellular trafficking, and neuronal resilience [14,16,17,25]. Consequently, developmental genetic perturbations may have biological consequences that extend far beyond circuit formation, influencing neuronal function and the capacity to respond to cumulative physiological stress across a lifetime.

These observations raise the possibility that early developmental perturbations may influence neuronal resilience decades after brain development is complete. Several conceptual frameworks have begun to address this possibility, proposing that neurodevelopment and aging should not be viewed as biologically independent processes but, instead, as interconnected phases of neuronal lifespan. Among these, Shabani and Hassan [26] proposed that subtle developmental abnormalities affecting neuronal maturation, developmental timing, and circuit assembly may generate an “at-risk normal brain” that initially functions within compensatory limits but progressively loses resilience during aging. Within this framework, developmental alterations do not inevitably cause neurodegeneration but instead establish latent states of neuronal vulnerability that may interact with aging-associated cellular stressors over time.

Several cellular processes altered in ASD, including mitochondrial dysfunction, oxidative stress, impaired proteostasis, calcium dysregulation, chronic neuroimmune activation, circadian disruption, and intracellular trafficking abnormalities, have also been implicated in age-related neurodegenerative disorders [27,28,29,30,31,32,33,34,35]. However, the biological consequences of these alterations depend on developmental stage, neuronal subtype, compensatory capacity, and aging context. For example, whereas ASD has frequently been associated with altered synaptic development and impaired synaptic pruning, Alzheimer’s disease is characterized by progressive synapse loss during aging. Accordingly, the overlap between ASD and neurodegenerative disorders should be interpreted as reflecting shared cellular mechanisms rather than a direct pathogenic trajectory linking these conditions.

Addressing these questions requires experimental models that integrate genetics, neuronal function, behavior, and aging into a single biological system. Among available model organisms, *Drosophila melanogaster* is uniquely suited to address these questions because it allows investigators to examine the consequences of developmental genetic perturbations continuously from embryogenesis through physiological aging within an identical genetic background. Approximately 75% of human disease-associated genes have recognizable fly orthologs [36,37,38], and fundamental pathways regulating synaptic transmission, calcium homeostasis [39], mitochondrial biology [40], circadian regulation [41], oxidative stress responses [42], and intracellular trafficking are highly conserved between flies and mammals. Combined with sophisticated genetic tools, a short generation time, and a broad repertoire of well-established behavioral paradigms, these features make *Drosophila* an ideal experimental system for investigating how developmental perturbations influence neuronal function throughout life [38,43,44].

In this review, I examine evidence that many ASD-associated genes function not only during neurodevelopment but also remain active in regulating neuronal physiology in adulthood and aging. Using *Drosophila* as an experimental and conceptual framework, I discuss how genetically diverse forms of ASD converge on conserved cellular pathways that influence neuronal function over time. Finally, I consider how this life-course perspective may help explain the relationship between early neurodevelopmental alterations, aging-related processes, and persistent neuronal vulnerability.

## 2. Lifelong Neuronal Vulnerability in ASD

A growing body of evidence suggests that ASD-associated developmental abnormalities may generate persistent states of neuronal vulnerability that extend far beyond early neurodevelopment [45,46]. In this review, I use the term “lifelong neuronal vulnerability” to emphasize that the proposed model refers to the long-term biological consequences of developmental perturbations on neuronal function across the lifespan, distinct from progressive neuronal degeneration or disease susceptibility alone [46]. Accordingly, neuronal circuits may remain functionally compensated during childhood and early adulthood, but may progressively lose their capacity to adapt to cumulative physiological challenges throughout the aging process [47,48].

This model does not imply that ASD inevitably progresses to neurodegeneration. Instead, it proposes that numerous ASD-associated molecular pathways remain active across the lifespan, regulating synaptic organization, cellular metabolism, mitochondrial dynamics, and intracellular signaling. Disruption of these homeostatic pathways during development may create a latent neuronal vulnerability that influences brain aging independently of classical neurodegenerative pathways.

**Figure 1:**
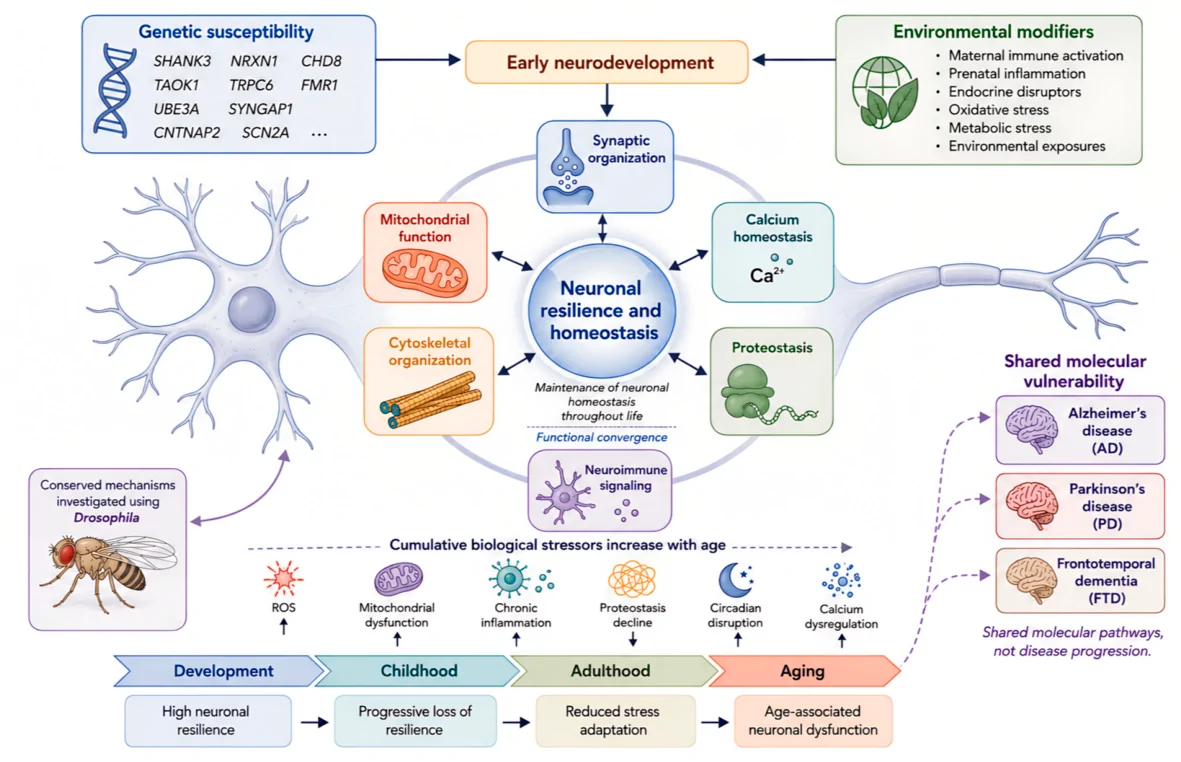
Conceptual model of lifelong neuronal vulnerability in autism spectrum disorder. Genetic susceptibility and environmental modifiers converge during early neurodevelopment to establish persistent alterations in neuronal resilience and homeostasis. Although diverse ASD-associated genes participate in distinct biological processes, they functionally converge on a limited number of conserved cellular pathways, including synaptic organization, mitochondrial function, proteostasis, calcium homeostasis, neuroimmune signaling, and cytoskeletal organization. Throughout life, these pathways regulate neuronal maintenance and progressively interact with cumulative biological stressors, such as oxidative stress, mitochondrial dysfunction, chronic inflammation, circadian disruption, and calcium dysregulation, leading to a gradual decline in neuronal resilience and increased vulnerability to age-associated neuronal dysfunction. This model proposes shared molecular mechanisms that may influence vulnerability to late-life neurological disorders without implying a direct causal progression from ASD to neurodegenerative disease. These conserved pathways can be modeled and investigated using *Drosophila* as a powerful experimental system. Solid arrows indicate the direction of the proposed relationships, bidirectional arrows indicate reciprocal interactions between cellular processes, and dashed arrows indicate shared molecular vulnerability rather than disease progression. Colors are used for visual organization and do not represent quantitative values.

This perspective provides a conceptual bridge between neurodevelopment and aging, recognizing that conserved cellular pathways modulate neuronal function throughout the lifespan while producing distinct biological and clinical outcomes shaped by developmental stage, genetic background, and environmental context. These shared mechanisms, encompassing mitochondrial function, proteostasis, neuroimmune signaling, calcium homeostasis, and synaptic regulation, may therefore shape the long-term trajectory of neuronal function and its response to age-associated stress [49] (Figure 1).

### 2.1. Functional Convergence of ASD-Associated Genes on Neuronal Resilience

Although ASD is among the most genetically heterogeneous neurodevelopmental disorders, its numerous risk genes converge on a limited repertoire of conserved cellular pathways rather than driving independent pathogenic mechanisms [16,17,18,19]. These include synaptic organization, calcium homeostasis, proteostasis, mitochondrial function, neuroimmune communication, cytoskeletal dynamics, and intracellular trafficking, all of which are essential for sustaining neuronal physiology [4,14,16,17]. Because these processes remain active beyond development, genetic perturbations associated with ASD can influence neuronal function throughout life rather than operating exclusively during circuit formation [25,50].

This functional convergence explains why genetically distinct forms of ASD frequently produce similar cellular phenotypes despite their heterogeneous etiologies [15,19]. Rather than acting independently, ASD-associated proteins form interconnected molecular networks in which the disruption of different components can alter common aspects of neuronal biology, including synaptic transmission, network excitability, cellular metabolism, and adaptive responses to physiological stress [16,18]. Notably, these same pathways are compromised in aging and several neurological disorders, particularly those involving impaired proteostasis, mitochondrial dysfunction, oxidative stress, and chronic neuroimmune activation [51,52,53,54,55,56]. This overlap highlights how diverse genetic causes can compromise core biological functions that become increasingly critical for maintaining neuronal performance with age.

Selected ASD-associated genes discussed throughout this review, together with their principal biological functions and conserved *Drosophila* orthologs, are summarized in Table 1.

#### 2.1.1. Synaptic Organization

Synaptic dysfunction is one of the most consistent biological features of ASD and represents a major point of convergence among genetically diverse forms of the disorder [16,18,60]. The establishment and maintenance of functional synapses rely on coordinated interactions between presynaptic adhesion molecules, postsynaptic scaffold proteins, and signaling complexes that regulate neurotransmitter release and activity-dependent plasticity [54,57]. Disruption of these molecular interactions alters neuronal connectivity and the balance between excitatory and inhibitory neurotransmission, a hallmark of ASD.

Among the best characterized synaptic proteins are the neurexins (NRXN1–3), presynaptic adhesion molecules that interact with postsynaptic neuroligins (NLGN1–4) to establish synapse identity and regulate neurotransmitter release, synaptic specificity, and excitatory/inhibitory (E/I) balance [57]. Rare variants affecting *NRXN1*, *NLGN3*, and *NLGN4* genes are well-established genetic causes of ASD and intellectual disability [59,85]. Experimental models further demonstrate that disruption of neurexin-neuroligin signaling impairs glutamatergic and GABAergic transmission, alters network synchronization, and disrupts activity-dependent synaptic plasticity, leading to behavioral abnormalities relevant to ASD [86,87,88,89].

Postsynaptic organization is largely coordinated by the SHANK (SHANK1-3) family of scaffold proteins, which assemble glutamate receptors, signaling complexes and cytoskeletal components within the postsynaptic density [90]. For example, loss of SHANK3 disrupts AMPA and NMDA receptor organization, compromises long-term synaptic plasticity and dendritic spine maturation, and alters corticostriatal circuitry, resulting in deficits in social behavior and increased repetitive behaviors in both ASD patients and experimental models [60,61,91,92,93,94].

Although these proteins perform distinct molecular functions, they cooperatively sustain synaptic organization and circuit function. Because synaptic adhesion molecules and scaffold proteins remain essential for receptor trafficking, dendritic spine stability, and activity-dependent plasticity throughout adulthood, disruption of these systems can influence neuronal network dynamics well beyond early brain development.

#### 2.1.2. Translational Regulation and Proteostasis

Maintaining synaptic function relies on precise control of protein synthesis, folding, trafficking, and degradation. Because mature neurons are post-mitotic, they cannot clear damaged proteins through cell division; consequently, proteostasis is essential for sustaining neuronal physiology and activity-dependent plasticity [95,96,97,98,99].

One of the best-characterized regulators of neuronal translation is the Fragile X Messenger Ribonucleoprotein (FMRP), encoded by the *FMR1* gene. FMRP is an RNA-binding protein that represses the local translation of synaptic mRNAs involved in AMPA receptor trafficking, actin cytoskeleton remodeling, calcium signaling, ion channel regulation, and synaptic plasticity [32,63]. Under physiological conditions, FMRP restricts excessive protein synthesis downstream of group I metabotropic glutamate receptor (mGluR) activation, thereby supporting dendritic spine maturation and synaptic function [100]. Loss of FMRP results in exaggerated activity-dependent translation, immature dendritic spines, cortical hyperexcitability, impaired synaptic plasticity, cognitive dysfunction, and sleep abnormalities, features that characterize Fragile X syndrome, the most common monogenic cause of ASD [62,101,102,103].

Protein homeostasis also depends on efficient degradation of damaged or unnecessary proteins. A central regulator of this process is the E3 ubiquitin ligase UBE3A, which controls ubiquitin-mediated protein degradation, synaptic remodeling, and protein turnover [64,104]. Maternal loss of *UBE3A* causes Angelman syndrome, whereas increased gene dosage has been associated with ASD [65,105]. Disruption of UBE3A-mediated ubiquitination alters synaptic receptor turnover and autophagic flux, promoting abnormal protein accumulation and defective synaptic remodeling [106,107]. Beyond UBE3A, several ASD-associated genes converge on proteostatic pathways involving the ubiquitin-proteasome system, autophagy, endosomal trafficking, and mTOR-dependent translational regulation [98,108,109,110].

Together, FMRP, UBE3A, and related proteostatic pathways ensure that neurons maintain a functional proteome capable of supporting synaptic transmission and plasticity. Disruption of these distinct mechanisms of protein synthesis and degradation can converge on similar defects in synaptic function [111].

#### 2.1.3. Mitochondrial Dysfunction and Oxidative Stress

Mitochondrial dysfunction is one of the most frequently reported biological alterations in ASD. Studies in both patients and experimental models have identified reduced oxidative phosphorylation, impaired ATP synthesis, abnormal electron transport chain (ETC) activity, altered mitochondrial morphology, and increased reactive oxygen species (ROS) production, even without classical mitochondrial disease [112,113,114,115,116,117,118]. These findings indicate that subtle bioenergetic defects can influence neuronal physiology.

Neurons are particularly sensitive to mitochondrial dysfunction because of their high energy demands and limited glycolytic capacity. Deficiencies in ETC complex I and complex IV, frequently reported in ASD, impair ATP production, synaptic transmission, axonal transport, calcium buffering, and activity-dependent plasticity [115,116,119]. In addition to ATP synthesis, mitochondria regulate redox signaling, apoptosis, neurotransmitter release, and neuronal differentiation, demonstrating that mitochondrial integrity underpins multiple pathways critical for lifelong neuronal function [120].

Oxidative stress is a major consequence of mitochondrial dysfunction [121,122,123]. Although ROS normally participate in intracellular signaling, chronic ROS accumulation promotes mitochondrial DNA damage, lipid peroxidation, protein oxidation, cytoskeletal destabilization, and synaptic dysfunction [124]. Consistent with this, ASD patients frequently exhibit elevated oxidative stress biomarkers together with reduced antioxidant defenses, including impaired glutathione metabolism and superoxide dismutase activity [125,126].

In addition to energy production, neurons rely on highly coordinated mitochondrial quality-control mechanisms to preserve organelle integrity throughout life. These mechanisms include mitochondrial fusion and fission, axonal transport, and selective elimination of damaged mitochondria through mitophagy, thereby ensuring an adequate supply of functional mitochondria to synaptic compartments with high metabolic demand. Among the principal regulators of this process, PTEN-induced kinase 1 (PINK1) and the E3 ubiquitin ligase, Parkin, are particularly important for neuronal mitochondrial quality control [72]. Under conditions of mitochondrial depolarization, PINK1 accumulates on the outer mitochondrial membrane and recruits Parkin, initiating ubiquitination of damaged mitochondrial proteins and subsequent autophagic clearance [72]. Defects in the PINK1/Parkin pathway impair mitochondrial turnover, leading to fragmentation, ATP depletion, oxidative stress, calcium dysregulation, and activation of pro-apoptotic signaling [127,128]. The importance of these mechanisms has been demonstrated extensively in *Drosophila*, where disruption of either PINK1 or Parkin causes progressive mitochondrial abnormalities, locomotor impairment, dopaminergic neuron loss, increased oxidative stress, and reduced lifespan [129,130,131].

Although relatively few ASD-associated genes encode mitochondrial proteins directly, recent systems-level studies demonstrate that mitochondrial dysfunction frequently emerges as a downstream consequence of genetically distinct neurodevelopmental variants [132]. Proteomic analyses in isogenic human cortical organoids carrying independent ASD-associated copy number variants revealed similar alterations in mitochondrial protein networks, with particularly strong convergence on organelle translation. These findings suggest that mitochondrial dysfunction is not strictly restricted to defects in mitochondrial genes themselves but instead represents a common cellular response to diverse genetic insults that converge on mitochondrial metabolism and protein homeostasis.

#### 2.1.4. Neuroimmune Signaling and Chronic Inflammation

Neuroimmune signaling is now recognized as an essential component of neuronal physiology, not a system activated exclusively during infection or tissue injury [133,134,135]. In addition to host defense, immune signaling regulates synapse formation, circuit refinement, neuronal plasticity, and tissue homeostasis, expanding its role far beyond classical inflammatory responses.

Among the cellular mediators of these interactions, microglia occupy a central position. In the healthy brain, they continuously survey the neural environment and participate in synapse formation, axonal remodeling, extracellular matrix organization, and activity-dependent plasticity [133,136,137]. During development, microglia eliminate unnecessary synapses while preserving functionally relevant connections, thereby refining neuronal circuits in response to neuronal activity. This function persists in the adult brain, where microglia continue to shape synaptic remodeling and tissue homeostasis.

A key mechanism underlying circuit refinement involves the classical complement cascade. Complement proteins such as C1q and C3 selectively tag weak or inactive synapses for elimination through complement receptor 3 (CR3)-dependent microglial phagocytosis [137,138]. Precise regulation of this pathway is essential for establishing functional neuronal connectivity, whereas excessive or insufficient pruning alters circuit organization and excitatory-inhibitory balance. Accordingly, disrupted complement signaling and aberrant microglial pruning are linked to the atypical cortical connectivity that characterizes ASD [139,140].

Persistent immune activation also influences several aspects of neuronal physiology. Activated microglia release pro-inflammatory cytokines, reactive oxygen species (ROS), nitric oxide, and excitatory amino acids that alter mitochondrial metabolism, calcium signaling, and protein turnover in neighboring neurons [141]. Similar alterations have been described in ASD, where sustained inflammatory signaling is accompanied by impaired astrocytic metabolic support, defective glutamate clearance, blood–brain barrier dysfunction, and increased oxidative stress [28,142].

Age further modifies these interactions through the gradual development of inflammaging, a state of chronic low-grade inflammation associated with immune senescence and sustained activation of innate immune pathways [143,144]. Aging microglia acquire a phenotype characterized by exaggerated cytokine production, reduced phagocytic precision, mitochondrial dysfunction, and diminished metabolic flexibility [133,145]. Consistent with this, developmental mechanisms that normally regulate circuit refinement, including complement-mediated synaptic pruning, become aberrantly reactivated in disorders such as Alzheimer’s disease, where excessive complement activation contributes to early synapse loss [146,147].

Although inflammatory alterations have been consistently reported in ASD, their precise contribution to disease pathophysiology remains unresolved [132]. Current evidence instead supports a dynamic interaction between immune signaling and neuronal physiology, in which inflammatory pathways continuously influence synaptic remodeling, cellular metabolism, and neuronal communication under both physiological and pathological conditions [133,134,148].

#### 2.1.5. Circadian Regulation and Sleep

Circadian rhythms synchronize neuronal and behavioral activity with cellular metabolism, endocrine signaling, and immune function through highly conserved molecular clock mechanisms [149,150]. Thus, these oscillatory networks regulate sleep, energy utilization, synaptic plasticity, protein turnover, antioxidant defenses, and DNA repair, enabling neuronal physiology to adapt dynamically to changing functional demands.

Sleep and circadian disturbances are among the most common comorbidities in ASD, affecting approximately 50–80% of autistic individuals [151,152]. Increasing evidence indicates that altered sleep architecture, circadian timing, and hormonal rhythmicity represent intrinsic components of ASD biology rather than secondary behavioral manifestations [149]. Consistent with this, several ASD-associated genes participate directly in circadian regulation. FMR1 modulates the translation of clock-related transcripts and contributes to sleep-dependent synaptic remodeling [153,154], whereas neurexins, neuroligins, SHANK proteins, and calcium-dependent signaling regulate forms of synaptic plasticity that fluctuate across the circadian cycle [155]. Accordingly, experimental ASD models consistently exhibit altered sleep architecture, disrupted circadian entrainment, and impaired rhythmic gene expression [156].

Sleep also provides a physiological state that supports synaptic renormalization, metabolic recovery, and the clearance of potentially toxic metabolites [157]. Chronic sleep disruption compromises these processes while impairing mitochondrial function, ATP production, antioxidant defenses, autophagy, and inflammatory regulation [149,158]. Instead of being an independent pathogenic mechanism, circadian disruption may act to exacerbate cellular abnormalities already present in ASD.

Age-related weakening of circadian rhythms further underscores the necessity of biological timing for brain integrity. Reduced circadian amplitude, impaired melatonin signaling, altered microglial rhythmicity, and progressive sleep fragmentation accompany normal aging and represent early features of Alzheimer’s and Parkinson’s diseases [149,159]. Whether sleep disturbances initiate neuronal dysfunction or accelerate existing pathology is poorly understood. However, their close association with multiple cellular processes discussed throughout this review underscores the importance of circadian regulation in maintaining normal brain physiology.

#### 2.1.6. Cytoskeletal Dynamics and Intracellular Trafficking

Neuronal function depends on the continuous remodeling of the cytoskeleton and intracellular trafficking systems that coordinate organelle positioning, receptor recycling, vesicular and organelle delivery, mitochondrial transport, and dendritic architecture [160]. Given the highly polarized morphology of neurons, efficient transport is indispensable to sustain communication between distant cellular compartments and support local synaptic activity.

Numerous ASD-associated genes participate in microtubule dynamics, actin remodeling, vesicular trafficking, and axonal transport [17,161]. Among them, the TAO kinase family, particularly TAOK1 and TAOK2, has emerged as a key regulator of neuronal polarity, dendritic arborization, axonal growth, microtubule organization, and MAP kinase signaling [162,163,164,165]. Pathogenic variants in these kinases are associated with ASD, intellectual disability, developmental delay, and behavioral abnormalities [75,76]. Beyond their structural functions, TAOK kinases activate c-Jun N-terminal kinase (JNK), thereby linking cytoskeletal remodeling to stress signaling, apoptosis, and tau phosphorylation [165,166]. This dual role highlights the intricate interplay between structural organization and intracellular signaling in maintaining neuronal function.

The cytoskeleton also provides the microtubular networks along which kinesin- and dynein-dependent motor complexes transport synaptic vesicles, mitochondria, lysosomes, RNA granules, and signaling molecules throughout the neuron [167]. Efficient trafficking ensures the appropriate distribution of energy supplies, signaling proteins, and membrane components required for synaptic activity. Accordingly, abnormalities in dendritic spine morphology, neurite complexity, and axonal connectivity constitute hallmark phenotypes across diverse ASD models [168,169].

Disruption of intracellular transport compromises the delivery of essential cellular components long before overt neuronal degeneration becomes apparent. Impaired mitochondrial motility, defective receptor trafficking, and altered vesicular transport reduce ATP availability at synapses, disturb calcium buffering, and impair local signaling [170]. Similar trafficking defects have been described across several neurodegenerative disorders, underscoring the importance of cytoskeletal integrity for maintaining neuronal architecture and intracellular communication [171,172,173,174].

#### 2.1.7. Selective Neuronal Vulnerability

A fundamental question in neuroscience concerns the mechanistic basis of differential vulnerability across brain regions, specifically why some neurons degenerate while others survive despite sharing an identical genome. Current literature suggests that this selective vulnerability is shaped not by an isolated pathogenic event, but by development and intrinsic physiological properties, including metabolic demand, calcium homeostasis, synaptic activity, axonal architecture, and proteostatic capacity [26,175].

This differential vulnerability raises an intriguing hypothesis for ASD, a condition in which many risk genes govern critical steps in neuronal differentiation, circuit assembly, synaptic maturation, and critical-period plasticity [15,17,175]. Alterations in these programs do not necessarily produce immediate pathology but can subtly modify neuronal metabolism, connectivity, and stress responses, thereby influencing how neurons adapt to homeostatic challenges later in life [26,176].

Human cortical development exemplifies this principle. Compared with other mammals, prolonged neurogenesis, delayed cortical maturation, and extended periods of synaptic refinement provide exceptional opportunities for circuit specialization while simultaneously increasing the window during which genetic and environmental factors can influence brain organization [15,177].

Recent studies indicate that extending neurogenic programs contributes directly to neocortical expansion by prolonging neural progenitor maintenance and neuronal production [178,179,180]. Association cortices responsible for executive function, social cognition, and higher-order integration are among the last regions to mature and remain highly metabolically active throughout life, characteristics that may increase their susceptibility to dysfunction [181,182,183,184].

Rather than acting independently, the cellular processes discussed throughout this review, including synaptic organization, proteostasis, mitochondrial function, neuroimmune signaling, circadian regulation, and intracellular trafficking, collectively define the physiological capacity of neurons to maintain normal function. Differences in how neuronal populations integrate these processes ultimately determine their selective vulnerability under both developmental and age-related challenges. Dopaminergic neurons, for example, are particularly sensitive because of their sustained calcium influx and high oxidative burden, whereas long-range cortical projection neurons rely heavily on mitochondrial function and efficient intracellular transport [185].

Viewed from this perspective, selective neuronal vulnerability emerges as a unifying biological concept to interpret genetically diverse forms of ASD. It emphasizes that early alterations in neuronal development set a baseline of intrinsic properties that influence how individual neuronal populations respond to cellular stress across the lifespan.

### 2.2. ASD Across the Lifetime

Although historically categorized as a childhood neurodevelopmental condition, the growing demographic of autistic individuals reaching older adulthood has redirected neurobiological research toward the biological processes that shape brain function over time [45,186].

Longitudinal cohorts reveal profound phenotypic heterogeneity among autistic adults, with trajectories of executive function, processing speed, memory, emotional regulation, sleep, and adaptive behavior varying considerably between individuals [6,187,188], suggesting that long-term outcomes do not follow a single biological trajectory. Instead, they likely reflect differences in how genetically determined neuronal properties interact with cumulative physiological demands over time.

Neuroendocrine dysregulation represents a plausible driver of this systemic variability. The hypothalamic-pituitary-adrenal (HPA) axis integrates endocrine, metabolic, circadian, and immune signaling, and altered cortisol dynamics have been reported in autistic cohorts [189,190]. Sustained glucocorticoid exposure disrupts mitochondrial bioenergetics and glucose utilization, impairs hippocampal plasticity, and accelerates oxidative damage [191]. In parallel, evidence from the BTBR mouse model indicates altered expression of DNA repair genes, including *Gadd45a*, *Ogg1*, and *Xrcc1* [192], which may destabilize long-term neuronal viability.

**Figure 2:**
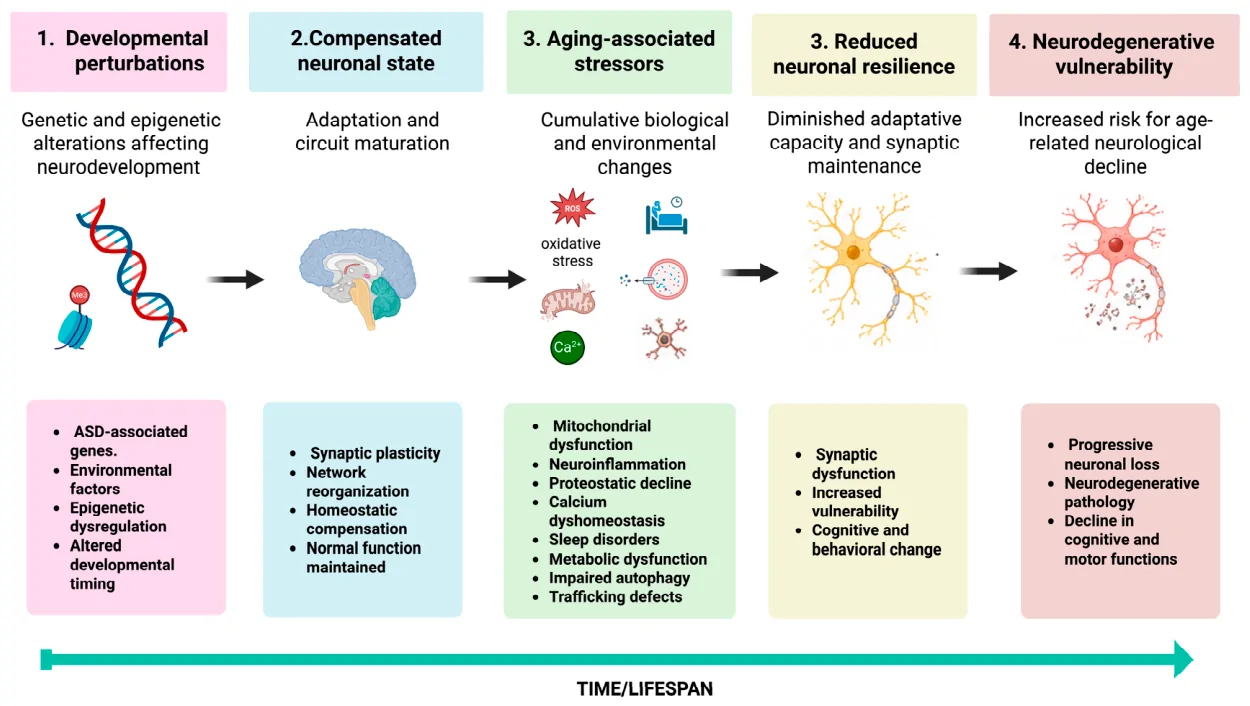
Conceptual framework of lifelong neuronal vulnerability in autism spectrum disorder. Schematic representation of how genetic, epigenetic, and environmental risk factors associated with ASD may shape neuronal physiology from development through aging. Early neurodevelopmental alterations establish distinct baseline cellular properties; while initially buffered by adaptive homeostatic mechanisms, these traits interact dynamically over time with cellular stressors, including mitochondrial dysfunction, oxidative stress, neuroimmune activation, circadian disruption, impaired proteostasis, intracellular trafficking defects, calcium dysregulation, autophagy decline, and reduced DNA repair capacity. The colored panels represent successive conceptual stages across the lifespan, whereas black arrows indicate a potential trajectory rather than an inevitable biological progression. The symbols illustrate representative molecular, cellular, and physiological processes associated with each stage, and the green arrow indicates increasing time across the lifespan. This model proposes that cumulative stressors may progressively reduce neuronal resilience and increase vulnerability to age-related neurological decline; however, it does not imply that all individuals with ASD will develop neurodegeneration. Created in BioRender. PALACIOS, A. (2026) https://app.biorender.com/citation/6a1cd50d809f0965de449e61 (accessed on 6 August 2026).

Ultimately, understanding how developmental programs establish cellular resilience offers a mechanistic model to explain the remarkable heterogeneity observed across adulthood and aging, while highlighting novel therapeutic targets to preserve lifelong brain health (Figure 2).

## 3. *Drosophila* as a Model for Lifelong Neuronal Vulnerability

### 3.1. Connecting Neurodevelopment and Brain Aging in a Single Experimental Model

**Figure 3:**
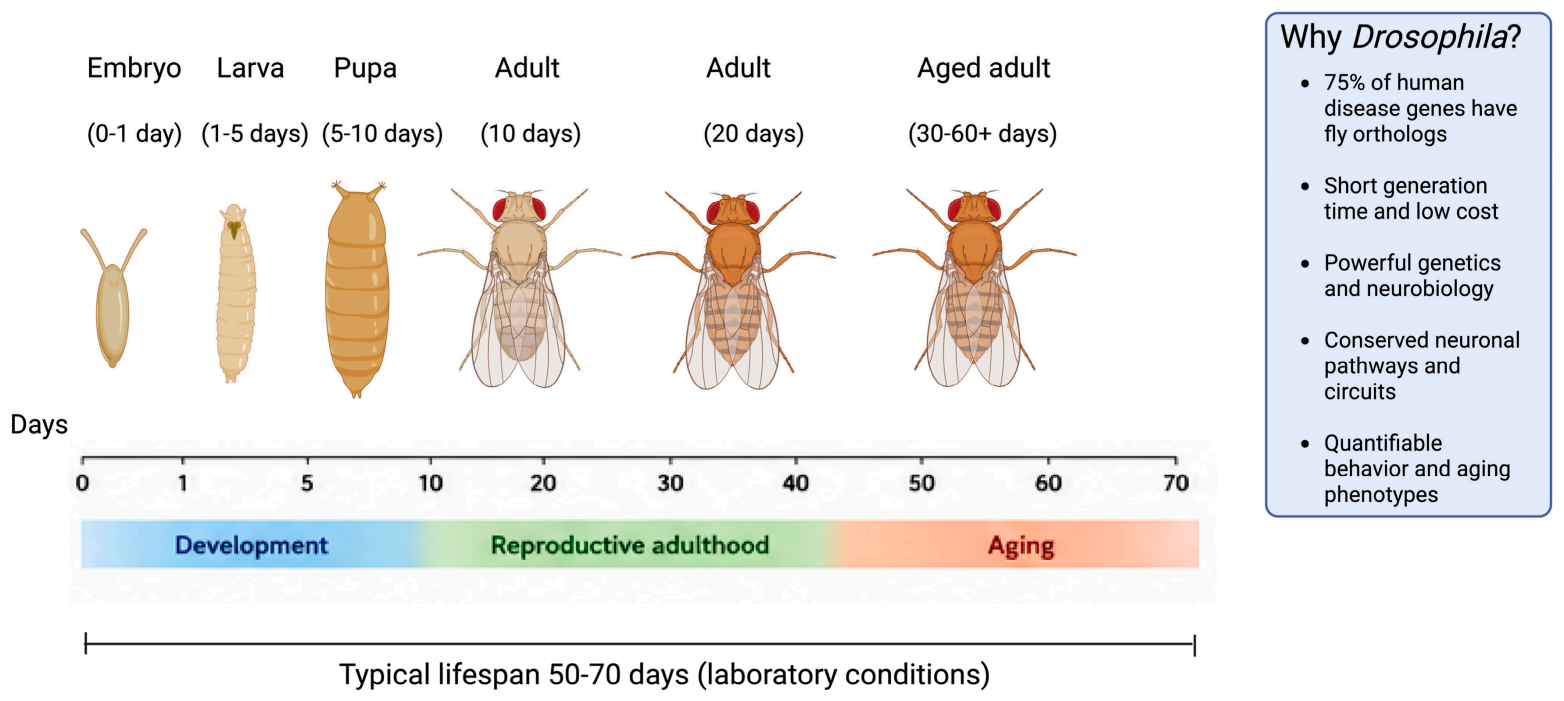
*Drosophila* life cycle and lifespan. *Drosophila* has a rapid and well-defined life cycle that permits neurodevelopment, adulthood, and aging to be examined within a short experimental period. Under standard laboratory conditions, embryonic and larval development is followed by pupation, with adult emergence occurring approximately 10 days after egg laying. Adult flies can then be studied during young adulthood, reproductive maturity, and advanced age. Lifespan commonly ranges from approximately 50 to 70 days at 25 °C, although longevity varies with genotype, diet, sex, and environmental conditions. This compressed life history supports age-stratified analyses of neuronal function, behavior, mitochondrial physiology, proteostasis, sleep, circadian regulation, and neurodegenerative phenotypes. The arrow indicates the temporal progression through the *Drosophila* life cycle and adult lifespan. Colors distinguish developmental and adult life stages and do not represent quantitative values or experimental conditions. Created with BioRender. PALACIOS, A. (2026) https://BioRender.com/lg83xv8 (accessed on 6 August 2026).

An important challenge is understanding how genetic alterations influence neural function across the lifespan. *Drosophila* combines a rapid life cycle with conserved neuronal pathways, precise genetic manipulation, and quantifiable behavioral phenotypes, making it particularly suitable for studying ASD-associated mechanisms over time [193] (Figure 3).

Although the fly brain is anatomically simpler than the mammalian nervous system, many mechanisms regulating synaptic transmission, mitochondrial function, intracellular trafficking, calcium signaling, proteostasis, circadian rhythms, sleep, and innate immune responses are conserved [194]. Approximately 75% of human disease-associated genes have recognizable fly orthologs, including numerous genes implicated in ASD, intellectual disability, epilepsy, and neurodegenerative disorders [36,38,195] (Table 1).

The short lifespan of *Drosophila* makes it possible to compare developmental, adult, and age-dependent phenotypes within weeks and in the same genetic background. This temporal continuity allows investigators to determine whether the effects of an ASD-associated variant are restricted to circuit formation, persist into adulthood, or become more evident as physiological function declines with age [36].

Genetic accessibility further distinguishes the fly model. GAL4/UAS, LexA/LexAop, and Q systems permit cell- and stage-specific gene manipulation, while CRISPR/Cas9, RNA interference, and large-scale screening approaches support rapid functional analysis of disease-associated variants [196,197,198] (Table 2). These experimental capabilities are further strengthened by an extensive community infrastructure. FlyBase (flybase.org) [199] is a model organism database that provides comprehensive gene annotation, expression profiles, phenotypic datasets, and human ortholog predictions, while integrative resources such as the DRSC Integrative Ortholog Prediction Tool (DIOPT) [200] and MARRVEL [201] facilitate the identification and prioritization of conserved disease genes by linking human and model organism datasets. Public repositories, including the Bloomington Drosophila Stock Center (BDSC; Indiana University, USA) and the Vienna Drosophila Resource Center (VDRC; Vienna BioCenter), maintain extensive collections of mutant, GAL4, UAS, RNAi, CRISPR, and transgenic lines, enabling rapid functional interrogation of candidate genes and experimental reproducibility.

Behavioral analyses provide a direct link between molecular alterations and nervous system function. Fly circuits regulate sleep, circadian activity, locomotion, learning, memory, sensory processing, courtship, and social interaction, allowing researchers to follow the consequences of genetic manipulation across different stages of adult life [202]. Models of ASD-associated genes reproduce phenotypes such as altered social behavior, repetitive actions, sleep disruption, synaptic abnormalities, and impaired cognitive performance [203,204]. These assays are particularly informative when combined with temporally controlled gene expression, because they allow experimental distinction between developmental and adult requirements for the same gene.

Moreover, *Drosophila* research in neurodegeneration also provides relevant experimental context. Fly models expressing human Aβ, tau, α-synuclein, mutant huntingtin, or TDP-43 reproduce protein aggregation, mitochondrial defects, synaptic deterioration, and progressive behavioral impairment associated with Alzheimer’s disease, Parkinson’s disease, Huntington’s disease, and amyotrophic lateral sclerosis [193,195,205]. Their relevance to ASD lies in their ability to compare how shared cellular processes, such as proteostasis, mitochondrial quality control, intracellular transport, and synaptic regulation, are affected by genetic alterations acting at different life stages, without implying a direct pathological continuum between neurodevelopmental and neurodegenerative disorders.

### 3.2. Conserved ASD Mechanisms Revealed by Drosophila Models

Functional studies of diverse ASD-associated genes in *Drosophila* have revealed convergence on several conserved neuronal processes [202,203]. Studying these genes in a common experimental system has enabled the distinction between gene-specific phenotypes and biological processes shared across genetically distinct forms of ASD.

The *dfmr1* mutant remains one of the best-characterized ASD models in *Drosophila*. Loss of *dfmr1* produces synaptic overgrowth, deficits in learning and memory, repetitive grooming, sleep fragmentation, and circadian abnormalities [206,207]. Importantly, several of these phenotypes can be partially rescued pharmacologically, demonstrating that developmental defects in synaptic regulation remain amenable to functional intervention in adulthood [208].

Similar observations have emerged from studies of *TRPC6*, whose fly ortholog, *trpγ*, regulates neuronal calcium signaling. Loss of *trpγ* impairs social interaction, hyperactivity, and learning and memory, while age and sex further modulate behavioral responses. Pharmacological activation of TRPC6 signaling with hyperforin partially restores several of these phenotypes, supporting the use of *Drosophila* for evaluating candidate therapeutic strategies [209].

A similar pattern is observed for synaptic adhesion molecules. *Nrx-1* and neuroligin homologs regulate synapse formation, neurotransmitter release, and circuit stability [210,211]. Their disruption alters social behavior, repetitive grooming, synaptic organization, and sleep regulation [212,213,214]. More recently, ASD-associated *NLGN3* variants have been shown to produce variant-specific alterations in synaptic morphology and sleep behavior [215].

Rather than serving as isolated models of individual genes, these studies illustrate how *Drosophila* has helped identify conserved biological mechanisms shared across genetically heterogeneous forms of ASD. The following sections examine how fly models have contributed to understanding specific cellular processes, including mitochondrial homeostasis, sleep and circadian regulation, neuroimmune signaling, and gut–brain interactions, that influence neuronal function throughout life.

### 3.3. Mitochondrial Dysfunction in Drosophila Models of ASD

Mitochondrial dysfunction has emerged as a recurring phenotype across genetically diverse *Drosophila* models of ASD. Mutations affecting genes involved in synaptic signaling, calcium regulation, intracellular trafficking, and protein homeostasis frequently produce similar mitochondrial abnormalities, indicating that impaired mitochondrial homeostasis represents a convergent cellular response rather than a defect restricted to genes encoding mitochondrial proteins [27,203,216].

Seminal studies in *Drosophila* established the *PINK1/Parkin* pathway as a central regulator of mitochondrial quality control. Loss of either gene disrupts mitophagy, leading to mitochondrial fragmentation, ATP depletion, oxidative stress, dopaminergic neurodegeneration, locomotor impairment, and reduced lifespan [131,217]. Although *PINK1* and *Parkin* are not representative ASD genes, these studies established an experimental basis for investigating mitochondrial dysfunction in genetically diverse neurological disorders, including ASD.

*Drosophila* models of neurodevelopmental disorders have revealed alterations in conserved pathways involved in neuronal homeostasis and cellular stress responses [203].

A major advantage of *Drosophila* is the ability to visualize mitochondrial dynamics in vivo. Live imaging has shown that aging progressively impairs mitochondrial transport through deterioration of the cAMP/PKA/Kinesin-1 pathway, reducing organelle mobility and increasing susceptibility to oxidative damage [218]. Genetically encoded reporters, including mitoGFP, mitoTimer, ROS sensors, ATP biosensors, calcium indicators, and ultrastructural analyses by electron microscopy enable mitochondrial morphology, turnover, and function to be monitored with high spatial and temporal resolution throughout life [219].

### 3.4. Sleep and Circadian Biology in Drosophila Models of ASD

Sleep is one of the most robust behavioral phenotypes used to investigate ASD-associated genes in *Drosophila*. The high conservation of sleep architecture and circadian regulation between flies and mammals, together with automated behavioral monitoring, has made the fly an ideal system for examining how genetic alterations influence neuronal physiology over time [220].

Several ASD-associated fly models display reproducible abnormalities in sleep and circadian behavior. Loss of *fmr1* disrupts circadian rhythmicity, sleep consolidation, and activity-dependent synaptic recovery [206], whereas mutations affecting *Nrx-1* and neuroligin homologs alter sleep architecture, locomotor rhythmicity, and social behavior [212,214]. Moreover, these phenotypes can be followed longitudinally, allowing developmental alterations to be evaluated throughout adult life.

The development of the Drosophila Activity Monitoring (DAM) system enables automated quantification of sleep duration, fragmentation, locomotor activity, and circadian rhythmicity in large cohorts of flies [221]. Combined with calcium imaging, optogenetics, electrophysiology, and genetically encoded metabolic reporters, these approaches allow behavioral phenotypes to be directly linked to neuronal activity and cellular physiology.

Beyond behavioral analysis, fly models have shown that chronic sleep disruption and circadian dysfunction influence mitochondrial homeostasis, oxidative stress, proteostasis, autophagy, and lifespan [220,222]. These observations reinforce the concept that sleep disturbances in ASD extend beyond behavior, affecting conserved cellular processes already discussed throughout this review.

### 3.5. Neuroimmune Signaling and Gut-Brain Interactions in Drosophila Models of ASD

Although *Drosophila* lacks adaptive immunity, its conserved innate immune pathways, including Toll, Imd, JNK, JAK/STAT, and NF-κB-like signaling, provide a genetically tractable system for examining how immune signaling modifies neuronal function [223,224,225]. Genetic studies have shown that sustained activation of these pathways disrupts mitochondrial homeostasis, protein quality control, synaptic stability, and lifespan, whereas glial-specific immune activation is sufficient to alter sleep, locomotor behavior, and neuronal maintenance [226,227,228,229,230,231]. Among these pathways, JNK is particularly interesting because it integrates inflammatory signaling with oxidative stress, mitochondrial dysfunction, apoptosis, and cytoskeletal remodeling [232]. The convergence of TAOK1 and TAOK2 on JNK signaling further links ASD-associated developmental genes with cellular stress responses that remain active throughout adulthood [165].

The experimental versatility of *Drosophila* has also expanded the study of the gut–brain axis. Its simple microbiota, together with tissue-specific manipulation of intestinal epithelial cells and immune pathways, enables precise analysis of how peripheral immune and metabolic signals influence brain physiology [233,234]. Studies in flies have shown that microbiota-derived metabolites regulate mitochondrial metabolism, ROS signaling, insulin pathways, immune activity, and behavior, whereas age-associated dysbiosis promotes intestinal barrier dysfunction, chronic inflammation, metabolic imbalance, and cognitive decline [235,236,237]. Given the high prevalence of gastrointestinal dysfunction and altered microbiota composition in ASD [238], the ability to manipulate intestinal, microbial, immune, and neuronal components makes *Drosophila* a powerful experimental platform for investigating how gut-derived immune and metabolic signals influence neuronal physiology across the lifespan.

## 4. Future Directions and Therapeutic Perspectives

Future research should focus on identifying the molecular mechanisms that determine why some neuronal populations remain functionally resilient whereas others progressively lose adaptive capacity over time. Addressing this question will require closer integration of developmental neuroscience, aging biology, mitochondrial physiology, neuroimmunology, circadian biology, and systems neurogenetics. Furthermore, longitudinal approaches that combine single-cell and spatial transcriptomics, proteomics, metabolomics, epigenomics, and functional imaging will be particularly valuable for defining the cellular trajectories underlying neuronal resilience across the lifespan [176,239].

Another important challenge is the development of biomarkers capable of monitoring biological aging and neuronal resilience in ASD. Mitochondrial respiratory profiling, redox-sensitive biosensors, inflammatory signatures, DNA methylation clocks, circadian metrics, and neuroimaging-based measures of synaptic integrity may provide complementary approaches for stratifying long-term vulnerability and evaluating therapeutic responses [240,241,242]. Because ASD encompasses highly heterogeneous biological trajectories, integrated biomarker panels are likely to be more informative than individual molecular markers.

Progress in this field will also depend on combining complementary experimental models. No single system can fully capture the complexity of lifelong neuronal vulnerability. Human longitudinal cohorts, iPSC-derived neurons, cerebral organoids, mammalian models, and *Drosophila* each address distinct aspects of the problem. Human-derived systems provide access to species-specific developmental processes and cortical organization, whereas *Drosophila* offers unique advantages for lifespan studies, longitudinal behavioral analysis, rapid genetic screening, and mechanistic dissection of conserved molecular pathways [243].

This perspective also broadens therapeutic opportunities beyond the treatment of behavioral symptoms during childhood. Strategies aimed at preserving mitochondrial function, maintaining proteostasis, improving calcium homeostasis, regulating neuroimmune signaling, and stabilizing sleep and circadian rhythms may reduce the cumulative cellular stress associated with aging. Antioxidant therapies, modulation of mTOR signaling and autophagy, sleep-based interventions, and microbiota-targeted approaches have already shown encouraging results in preclinical studies [126,149,244,245]. In parallel, the experimental versatility of *Drosophila* continues to facilitate rapid genetic and pharmacological screening, providing an efficient platform for identifying candidate therapeutic targets before validation in mammalian systems.

## 5. Limitations and Outstanding Questions

Despite growing evidence linking ASD-associated pathways with aging, several fundamental questions remain unresolved. One of the most important is whether ASD increases susceptibility to classical neurodegenerative disorders or instead modifies neuronal resilience without necessarily leading to overt neurodegeneration. Current longitudinal and epidemiological data remain insufficient to distinguish between these possibilities.

A second challenge arises from the remarkable heterogeneity and broad phenotypic spectrum of ASD, where individuals carrying different, or even the same, variants can present with highly diverse clinical manifestations [12]. Different genetic backgrounds are likely to generate distinct trajectories of neuronal maintenance and aging, making it unlikely that all ASD-associated genes converge on identical long-term outcomes. Whether mutations affecting synaptic organization, mitochondrial function, translational regulation, chromatin remodeling, or intracellular trafficking produce common or divergent patterns of neuronal aging remains largely unknown.

Another unresolved issue concerns the temporal relationship between development and aging. Many cellular abnormalities observed in ASD may initially represent compensatory adaptations that preserve neuronal function during development but later become maladaptive under chronic metabolic, inflammatory, or oxidative stress. Defining when developmental adaptations transition into age-associated vulnerability remains a major challenge.

Experimental models also impose important limitations. Mammalian systems reproduce many aspects of cortical organization and higher cognitive function but are constrained by long generation times and experimental complexity. In contrast, *Drosophila* provides exceptional genetic accessibility and lifespan-scale analyses but cannot model human cortical expansion, prolonged developmental programs, language-associated circuitry, or higher-order cognition. Human organoids partially bridge this gap but remain limited by incomplete maturation and the absence of systemic physiological interactions.

## 6. Conclusions

Increasing evidence suggests that ASD involves persistent alterations in neuronal maintenance pathways extending far beyond early neurodevelopment. Many ASD-associated genes regulate mechanisms critically involved in synaptic stability, mitochondrial function, oxidative stress buffering, calcium homeostasis, intracellular trafficking, circadian regulation, proteostasis, and neuroimmune signaling. Importantly, these same pathways are also critically involved in aging and neurodegeneration. This overlap supports a broader framework in which developmental perturbations generate long-term states of neuronal vulnerability that progressively interact with metabolic, inflammatory, oxidative, and proteostatic stress throughout aging.

Within this context, *Drosophila* has emerged as a powerful translational platform for investigating conserved mechanisms linking neurodevelopment, aging, neuronal resilience, and neurodegeneration. Fly studies have identified age-dependent changes in behavior and neuronal function across several genetic models, providing a framework to investigate how developmental alterations influence neuronal physiology throughout life.

Future integration of developmental neuroscience, aging biology, systems neurogenetics, and lifespan neuroscience may fundamentally transform current understanding of ASD. Rather than viewing ASD exclusively as a static childhood condition, emerging evidence increasingly supports a dynamic model in which developmental and aging-associated processes remain deeply interconnected. Understanding these interactions may ultimately reveal novel strategies for preserving lifelong brain health and neuronal resilience. By providing experimental access to this continuum, *Drosophila* has become an important platform for exploring central questions in modern neuroscience, such as how early biological perturbations shape long-term trajectories of brain health and disease.

## Figures and Tables

**Table 1:** Representative ASD-associated genes discussed throughout this review, their conserved *Drosophila* orthologs, primary molecular functions, and biological processes contributing to lifelong neuronal resilience.

Biological Process	Human Gene	Drosophila Ortholog	FlyBase Annotation ID (CG)	Encoded Protein	Primary Molecular Function	Representative Reference(s)
Synaptic organization	*NRXN1*	*Nrx-1*	CG7050	Neurexin-1	Presynaptic cell adhesion molecule	[57,58]
Synaptic organization	*NLGN3*; *NLGN4*	*Nlg3*; *Nlg4*	CG34127; CG34139	Neuroligin-3; Neuroligin-4	Postsynaptic cell adhesion molecules	[57,59]
Synaptic organization	*SHANK3*	*Prosap*	CG30483	SHANK3	Postsynaptic scaffold protein	[60,61]
Translational regulation and proteostasis	*FMR1*	*Fmr1*	CG6203	Fragile X messenger ribonucleoprotein 1 (FMRP)	RNA-binding protein regulating local translation	[62,63]
Translational regulation and proteostasis	*UBE3A*	*Ube3a*	CG6190	UBE3A	E3 ubiquitin ligase	[64,65]
Ion channels and calcium homeostasis	*TRPC6*	*Trpγ*	CG5996	TRPC6	Calcium-permeable non-selective cation channel	[66]
Ion channels and calcium homeostasis	*SCN2A*	*para*	CG9907	Na_V_1.2	Voltage-gated sodium channel	[67,68]
Ion channels and calcium homeostasis	*CACNA1C*	*Ca-α1D*	CG4894	Ca_V_1.2	L-type voltage-gated calcium channel	[69,70]
Mitochondrial homeostasis	*PINK1*	*Pink1*	CG4523	PINK1	Serine/threonine kinase initiating mitophagy and mitochondrial quality control	[71,72]
Mitochondrial homeostasis	*PRKN*	*park*	CG10523	Parkin	E3 ubiquitin ligase mediating mitophagy and mitochondrial quality control	[73,74]
Cytoskeletal organization	*TAOK1*	*Tao*	CG14217	TAOK1	Serine/threonine kinase regulating microtubule dynamics	[75,76]
Circadian biology	*CLOCK*	*Clk*	CG7391	CLOCK	Circadian transcription factor	[77]
Circadian biology	*PER1*, *PER2*	*per*	CG2647	PER1; PER2	Core circadian clock proteins	[78,79]
Gene regulation	*MECP2*	*MBD-like*	CG8208	MeCP2	Methyl-CpG-binding transcriptional regulator	[80,81]
Gene regulation	*CHD8*	*kis*	CG3696	CHD8	ATP-dependent chromatin remodeler	[82,83]
Gene regulation	*DEAF1*	*Deaf1*	CG8567	DEAF1	Transcription factor	[84]

**Table 2:** *Drosophila* tools and experimental approaches for studying ASD and neuronal aging.

Tool/Approach	Description	Application in ASD Research	Applications in Aging Research
GAL4/UAS system	Tissue- and cell-type-specific expression of genes	Modeling gene overexpression or knockdown in neurons/glia and cell-type-specific expression of genes	Assess age-dependent effects in specific cell populations
RNA interference (RNAi)	Gene knockdown to reduce expression	Screening of ASD candidate genes and pathways	Identify genes involved in lifespan and age-related decline
CRISPR/Cas9 genome editing	Precise genome editing and mutant generation	Create loss-/gain-of-function models of ASD genes	Model age-associated genetic variants and modifiers
MARCM (clonal analysis)	Generation of marked clones for cell-autonomous studies	Study neuronal cell-autonomous defects	Analyze clonal survival and age-dependent vulnerability
Optogenetics	Light-controlled manipulation of neuronal activity	Assess circuit function and behavioral phenotypes	Test how aging alters circuit function and responsiveness
Behavioral assays	Quantifiable behavioral readouts	Social interaction, courtship, learning, memory, repetitive behaviors, sensory responses	Locomotor activity, sleep, cognition, age-related decline
Imaging techniques	Confocal, two-photon calcium imaging	Synaptic morphology, circuit structure, calcium dynamics	Monitor structural and functional changes across lifespan
Lifespan and stress paradigms	Lifespan analysis and controlled stress exposure	Test resilience to oxidative stress, seizures, inflammation	Identify factors that extend health span and resilience

## Data Availability

No new data were created or analyzed in this study. Data sharing is not applicable to this article.
